# Assessing the Effect of Damage and Steel Fiber Content on the Self-Sensing Ability of Coal Gangue-Cemented Composite by Electrochemical Impedance Spectroscopy (EIS)

**DOI:** 10.3390/ma18112467

**Published:** 2025-05-24

**Authors:** Meng Xiao, Feng Ju, Zequan He, Pai Ning, Tengfei Wang, Dong Wang

**Affiliations:** 1 State Key Laboratory of Intelligent Construction and Healthy Operation and Maintenance of Deep Underground Engineering, China University of Mining and Technology, Xuzhou 221116, China; m.xiao@cumt.edu.cn (M.X.); mechanics@cumt.edu.cn (D.W.); 2 School of Mechanics and Optoelectronic Physics, Anhui University of Science and Technology, Huainan 232002, China; hzq1993@cumt.edu.cn

**Keywords:** electrochemical impedance spectroscopy, EIS, coal gangue-cemented composites, self-sensing, equivalent circuits

## Abstract

Steel fibers (SFs) can form stable conductive networks in coal gangue-cemented composites (CGCCs), endowing CGCCs with excellent mechanical, electrical and self-sensing properties. Meanwhile, electrochemical impedance spectroscopy (EIS) provides a potential approach to evaluate the damage situation of SF-reinforced CGCC. In this paper, EIS responses of CGCCs with different SF content and damage levels were determined. An equivalent circuit was then explored, and the effect of the SF content and damage levels on its parameters was investigated. It was observed that CGCC with 0.8% SFs yielded the best result in terms of mechanical and self-sensing ability. In addition, damage such as microcracks primarily affects the conductive pathways induced by pores rather than those induced by SFs. More importantly, as a non-destructive method, the EIS technique is practical and promising for monitoring damage conditions of SF-reinforced CGCC in underground engineering.

## 1. Introduction

Coal gangue-cemented composites (CGCCs) have become a primary method for the disposal of coal gangue. In China, the annual generation of coal gangue (CG) ranges between 600 and 800 million tons, with a utilization rate of less than 70% [1]. The cumulative stockpile of CG has exceeded 7 billion tons and continues to grow by 300 to 350 million tons annually [2]. The extensive accumulation of CG poses serious environmental challenges, including land degradation, air and water pollution, and the risk of environmental and geological hazards, such as landslides, debris flows, and spontaneous combustion [3]. CGCCs have been widely applied in underground engineering, serving crucial roles in both backfill for mining operations and the construction of underground structures [4].

CGCCs provide essential support to the surrounding rock, playing a crucial role in maintaining the stability of underground spaces [5]. However, the high brittleness of concrete makes it prone to cracking over time due to complex environmental conditions. If damage, such as micro-cracks, is not promptly addressed, the service life of CGCC structures can be significantly reduced, potentially leading to catastrophic consequences [6]. Therefore, to ensure timely and long-term remediation of defects, in situ health monitoring of CGCC structures is necessary.

Intrinsic self-sensing cement-based sensors, enhanced by the incorporation of conductive fillers, form an electrically conductive network within the cementitious matrix [7,8,9,10,11,12]. This network not only improves electrical conductivity, crucial for self-sensing, but also enhances physical properties, such as reduced drying shrinkage and improved mechanical performance [13]. The development of such multifunctional concrete enables a wide range of sustainable and smart infrastructure applications [14]. These include self-sensing for structural health monitoring, thermal and electrical energy storage, electromagnetic interference shielding, and road deicing. Sun et al. [15] investigated the use of steel fibers (SFs) and steel wire (SW) in concrete, and their findings indicated that concrete incorporating both SFs and SW exhibited enhanced tensile mechanical properties and improved self-sensing capabilities. He et al. investigated the compaction characteristics of coal gangue backfilling material using the electrical resistivity method and discovered that the variation in stress closely aligns with changes in electrical parameters. Xiao et al. [16] examined the self-sensing capability of cement paste using the eddy current method, finding that SF-reinforced cement paste exhibited the highest strain gauge factor. The combination of improved behavior and natural compatibility with concrete structures renders these cement-based sensors preferable over extrinsic sensors for many applications, opening a door to in situ health monitoring of CGCC structures. Although laboratory research has been conducted on the stationary electrical behavior, mechanical properties, and stress/strain sensing capabilities of cement-based sensors, their effective application remains limited due to the lack of understanding of the sensing mechanism.

Electrochemical impedance spectroscopy (EIS) is a sensitive, non-destructive, and highly reproducible technique that is relatively easy to use [17,18,19,20,21]. It has been widely employed to characterize cementitious materials in various aspects, including early hydration, carbonation, dewatering, corrosion, permeability and durability, microstructural analysis, loading, cracking, and crack healing [22,23,24,25,26,27]. The study by Dong et al. [28] has shown that the carbonation resistance of fly ash-blended cement materials can be effectively evaluated using EIS, with the parameter of equivalent circuit serving as a key indicator of carbonation depth. Chi et al. [29] explored the hydration of cement with Na_2_CO_3_ using EIS, uncovering a correlation between microstructure, hydration products, and electrical resistivity, which supports the use of EIS as a potential alternative to conventional hydration testing methods. Almotlaq et al. [14] elucidate the ionic conduction mechanism in self-sensing concrete by EIS. Zhang et al. [22] investigated the piezoresistivity and piezopermittivity of self-sensing concrete under changing stress and moisture using the EIS method. The installation of electrodes in CGCC structures enables the application of EIS for monitoring structural damage in underground engineering environments. Consequently, systematic investigation of EIS response patterns under mechanical loading is critical to advancing damage assessment methodologies for these structures.

This study investigates the influence of conductive filler content and damage level on the self-sensing capability of SF-reinforced CGCCs (SF-CGCC) using the EIS method. SFs were chosen as conductive fillers, with five different SF content levels and a control group without SFs. Specimens with varying degrees of damage were prepared through load–unload experiments. EIS was employed to measure the electrical signal response of the SF-CGCC specimens. The self-sensing mechanism of SF-CGCC was analyzed using equivalent circuit fitting parameters and multiple regression analysis based on the EIS results.

## 2. Experimental Methodology

### 2.1. Raw Materials

**Figure 1:**
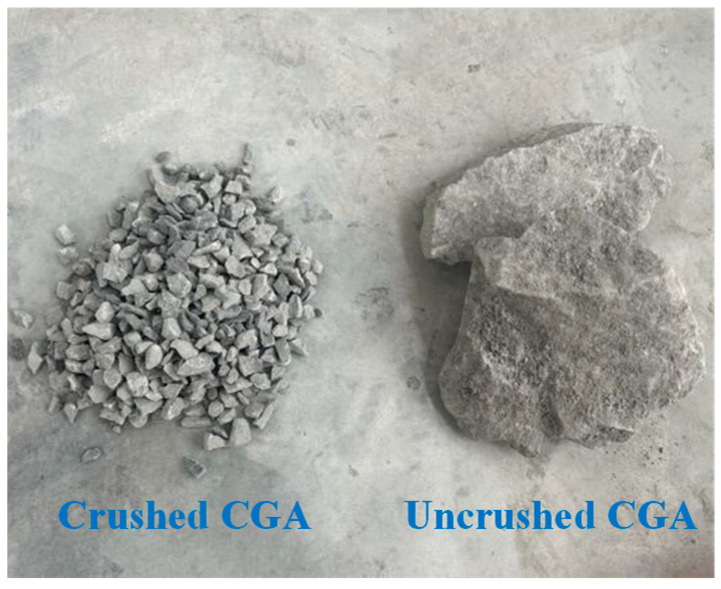
The crushed CGAs [30].

In this experiment, P.O 42.5 cement, produced by China United Cement Corporation, was used as the binder material. The aggregate consisted of coarse and fine CG aggregates (CGAs, as shown in Figure 1), sourced from Xuzhuang Coal Mine in Xuzhou City, Jiangsu Province, China.

**Figure 2:**
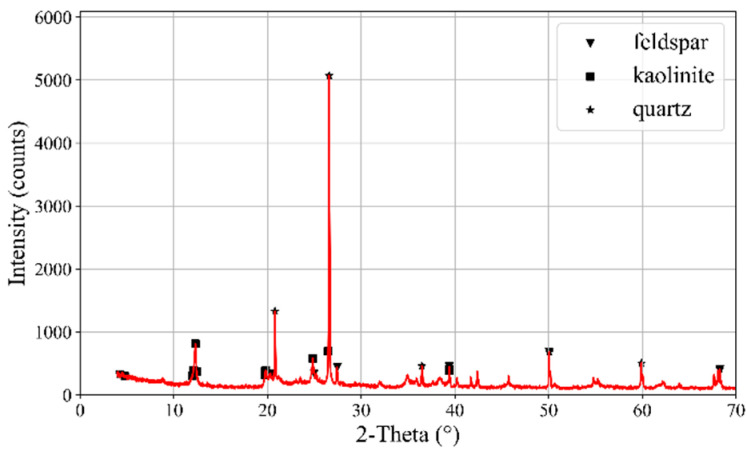
XRD pattern of CGAs [30].

**Figure 3:**
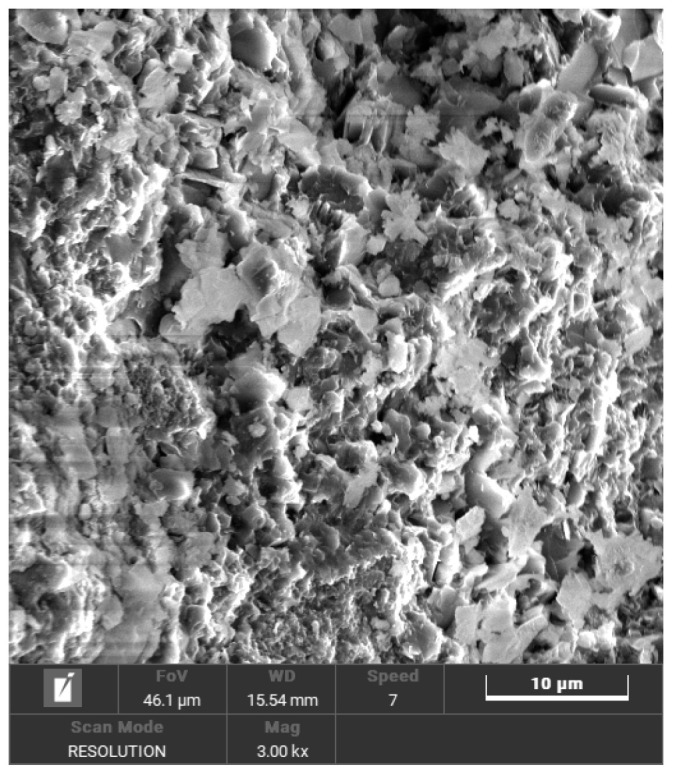
SEM image of CGAs.

**Figure 4:**
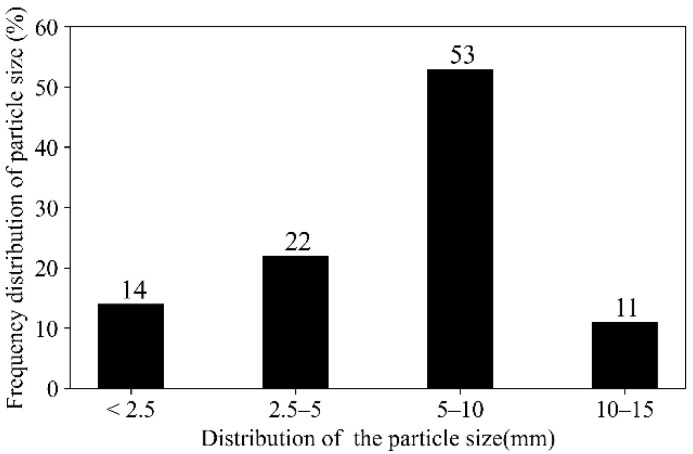
Particle distribution of the CGAs.

Elemental compositions of CGAs were determined via X-ray fluorescence spectroscopy (XRF; Bruker Tiger S8, Karlsruhe, Germany) equipped with a rhodium anode and a 75 μm Beryllium window. The system operated under a high-voltage power supply (4 kW, 60 kV, 170 mA) and utilized three interchangeable crystals (PET, XS-55, and LiF200) for multi-element detection across varying energy ranges. Mineralogical phases in CGAs were characterized using X-ray diffraction (XRD, Bruker D8, Karlsruhe, Germany) with Cu Kα radiation (30 mA, 40 kV). Scans were performed over a 2θ range of 5–70° with a step size of 0.02° and a dwell time of 0.02 s per step. The results are shown in Table 1 and Figure 2, respectively. XRF analysis revealed that the main components of the CGAs are SiO_2_ (62.113%) and Al_2_O_3_ (28.169%). The primary mineral phases identified in the CGAs include kaolinite, quartz, and feldspar. The microstructure of CGAs was investigated using an environmental scanning electron microscopy (SEM, Quanta 250, FEI, Hillsboro, OR, USA). Samples mounted on SEM stubs were analyzed under controlled operational parameters: acceleration voltage of 5–10 kV, probe current ranging from 1 pA to 2 mA, and a magnification level of 3000×. Figure 3 presents SEM images of the CGAs, revealing a porous surface texture. The physical properties of the CGAs were tested in accordance with ASTM-C127 standards [31], and the results are summarized in Table 2. The CGAs used in this study were produced by crushing large CG pieces using a jaw crusher (PEF60 × 100, Weiming, Ganzhou, China). The crushed CGA was then sieved into three distinct size ranges (<2.5 mm, 2.5 mm–5 mm, 5 mm–10 mm, and 10 mm–15 mm) using a square hole sieve. The grain size distribution is illustrated in Figure 4.

In addition, the SFs used in this study were 6 mm in length and had a diameter of 200 μm. The main mechanical properties reported by the manufacturer are tensile strength of 2060 MPa and elastic modulus of 200 GPa. Silica fume was incorporated to enhance the dispersion of the SFs, with a specific surface area of 19.5 m²/kg and a specific gravity of 0.65. A polycarboxylate superplasticizer produced by Feike Co., Ltd. (Foshan, China) was also used to improve fiber dispersion and enhance the workability of the mix. Tap water was used as the mixing water in this experiment.

### 2.2. Mix Proportion

SF-CGCC mixtures were prepared using a mix ratio of 1:0.4:0.04 (CGAs: cement: fume) by weight for all samples, with a constant water-to-cement ratio of 0.45. The slump of the cement mixture at the specified water-to-cement ratio was measured in accordance with the China national standard GB/T 50080-2016 [32], yielding a slump value of 230 mm. The SF-CGCC mixtures were produced by dispersing SFs into the mortar matrix at contents of 0% and 2.0% of the total volume. Based on trial experiments, the superplasticizer was added at 0.01% by weight of cement to ensure adequate workability without segregation. The mix design for all samples is presented in Table 3. Group S0.0 served as the reference, representing plain CGCC without SFs. Groups S0.4, S0.8, S1.2, S1.6, and S2.0 correspond to the SF content of 0.4%, 0.8%, 1.2%, 1.6%, and 2.0% by volume, respectively.

**Figure 5:**
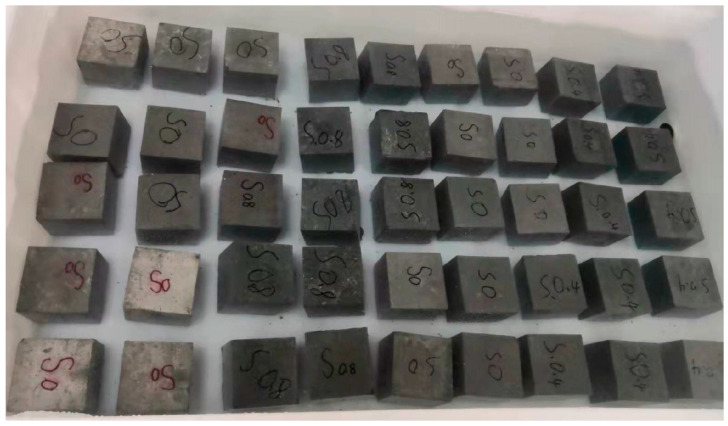
SF-CGCC samples prepared for mechanical test.

**Figure 6:**
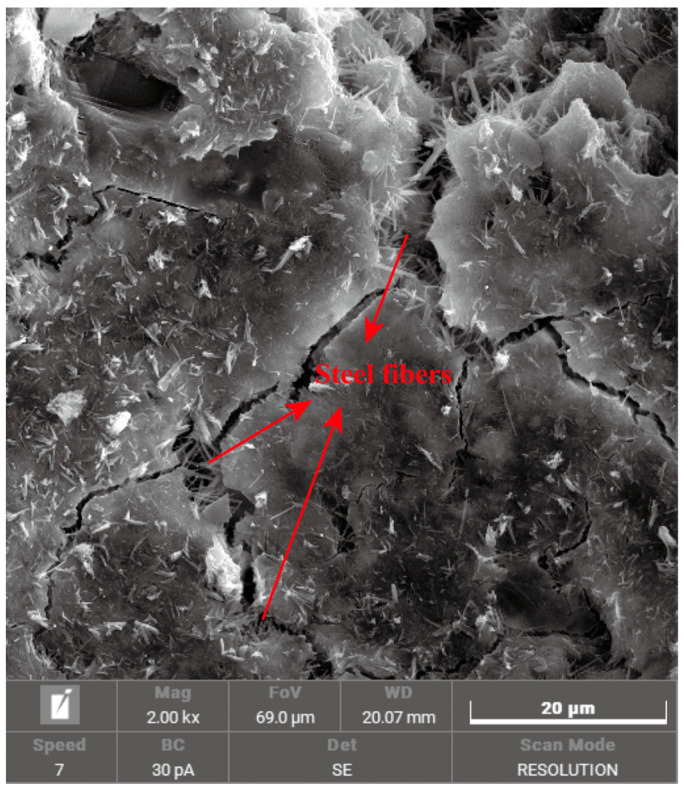
SEM images of SF-CGCC.

The SF-CGCC specimens were prepared using the following procedure: First, the pre-weighed SFs and silica fume were added into the mixer, followed by the addition of water and the water-reducing agent. The mixture was then mixed at low speed (25 r/min) for 60 s. Next, the cement was added to the mixer and blended at low speed (25 r/min) for 60 s, followed by high-speed (45 r/min) mixing for 120 s. After the mixture was fully homogenized, it was poured into oiled molds, with 18 samples of dimensions 100 × 100 × 100 mm cast at a time. The molds containing the fresh SF-CGCC were then vibrated for 60 s to remove air bubbles. The specimens were cured at 20 °C and 95% relative humidity for 24 h before demolding. Afterward, the specimens were submerged in water at room temperature for 28 days of curing, followed by air drying for 24 h (as shown in Figure 5). Finally, all the samples were drilled into cylinders with dimensions of Φ50 × 50 mm before testing. As shown in Figure 6, the SEM image of SF-CGCC reveals a homogeneous dispersion of SFs within the cement matrix, with no visible agglomeration observed at the fiber–matrix boundaries.

### 2.3. Testing Method

#### 2.3.1. Uniaxial Compressive Test and Initial Damage Preparation

The uniaxial compressive strength (UCS) tests were performed using a high-precision electro-hydraulic servo-controlled universal testing machine (WAW-2000; Keda Testing Machinery Co., Ltd., Changchun, China) with a maximum range of 2000 kN. The UCS tests were conducted using a load-controlled method with a loading rate of 0.45 kN/s. The UCS for each mixture was calculated as the average of three replicate samples.

To assess the effect of initial damage on the EIS of SF-CGCC, five different initial damage levels were designed under static compression: 0%*P*_max,s_, 50%*P*_max,s_, 60%*P*_max,s_, 70%*P*_max,s_, and 80%*P*_max,s_ (where *P*_max,s_ represents the maximum compressive load of SF-CGCC samples during UCS tests). The loading rate for these tests was also maintained at 0.45 kN/s.

#### 2.3.2. EIS Test

EIS is a nondestructive technique used to measure the resistance to electrical flow through connected nodes formed by moisture and conductive ions within materials. There are two types of EIS: potentiostatic and galvanostatic [26]. Potentiostatic EIS works by applying an alternating voltage signal across a wide frequency range and measuring the resulting current signal. In contrast, galvanostatic EIS measures the voltage signal while an alternating current is applied to the sample. The response to each driving frequency is expressed as impedance, represented as a set of complex numbers. The impedance spectrum, containing the information (voltage or current) for each frequency, is typically displayed in a Nyquist plot (showing the real and imaginary components of impedance) or a Bode plot (showing the magnitude and phase of impedance).

**Figure 7:**
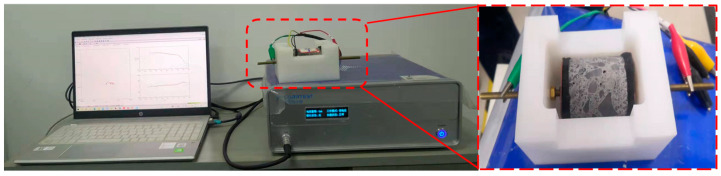
EIS testing machine and specific mold.

In this experiment, potentiostatic EIS was employed. The electrochemical workstation used was a Corrtest CS350H, Wuhan Corrtest Instruments Corp., Ltd., Wuhan, China (as illustrated in Figure 7), interfaced with a computer for data acquisition and recording. As recommended in references [15,29,33], the frequency range was from 1 MHz to 0.1 Hz, with a sinusoidal AC amplitude of 100 mV. Ten measurements were taken for each frequency range. Since the water content of the specimen significantly affects the impedance spectrum results, all specimens were conditioned at a constant temperature of 20 °C and 60% relative humidity for over 24 h prior to the impedance testing to eliminate the influence of moisture.

As shown in Figure 7, a custom mold was designed specifically for the EIS test. The electrode was made of stainless steel with a surface area matching that of the specimen’s base, while the rest of the mold was composed of insulating resin. During testing, a layer of wet sponge, which does not affect the impedance spectrum [34], was placed between the electrode and the specimen to ensure good contact between them.

## 3. Results and Discussion

### 3.1. Mechanical Properties

**Figure 8:**
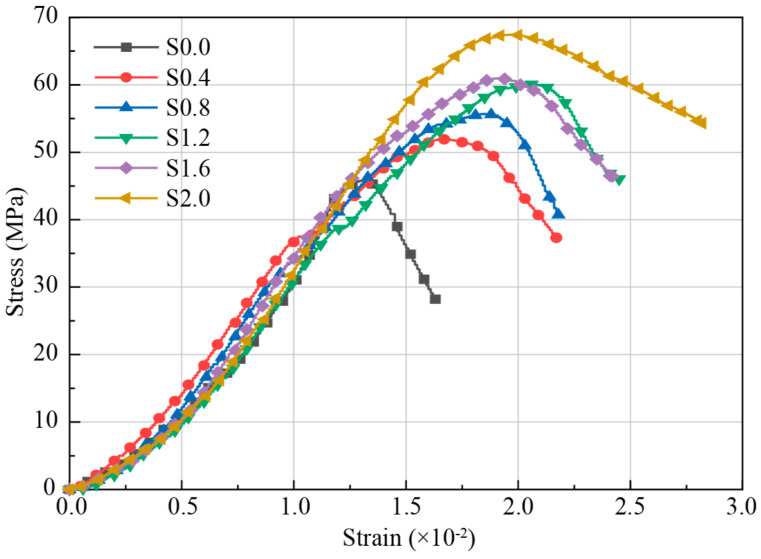
Stress–strain curves of samples with various SF contents.

Figure 8 illustrates the stress–strain curves under UCS for CGCC specimens with different SF content. As shown in Figure 8, during the elastic deformation phase, the stress–strain curves for specimens with varying amounts of SFs exhibit a high degree of similarity, suggesting that the addition of SFs has minimal effect on deformation in this phase. However, the post-peak behavior of curves is significantly influenced by the presence of SFs. Without SFs, the curve drops sharply after reaching peak stress, indicating a brittle failure of the specimen. With the addition of SFs, a noticeable non-linear section appears in the compression stress–strain curve. Beyond the elastic phase, the stress growth rate slows significantly compared to strain, as the specimen enters the plastic phase. During this phase, internal cracks begin to develop but do not yet form through-cracks. Approaching peak stress, the specimen enters a phase of stable crack development, where cracks merge, connect, and eventually grow into macroscopic fractures. At this point, SFs may be pulled out or even broken. After reaching peak stress, macroscopic cracks appear, and stress decreases rapidly while strain increases quickly. As the SF content increases, instances of fiber pull-out and breakage become more frequent, and the descending part of the stress–strain curve becomes progressively smoother [35,36]. It should be noted that the stress–strain curves of S1.2 and S1.6 exhibit comparable mechanical profiles. Specifically, the S1.6 specimen demonstrates a 1.5% higher compressive strength (60.9 MPa vs. 60.1 MPa) but an 8.4% lower peak strain (0.0190 vs. 0.0206) compared to S1.2. This inverse relationship between fiber content and ductility stems from excessive SFs agglomeration at 1.6% content, which generates localized stress concentrations and promotes brittle interfacial failure modes.

**Figure 9:**
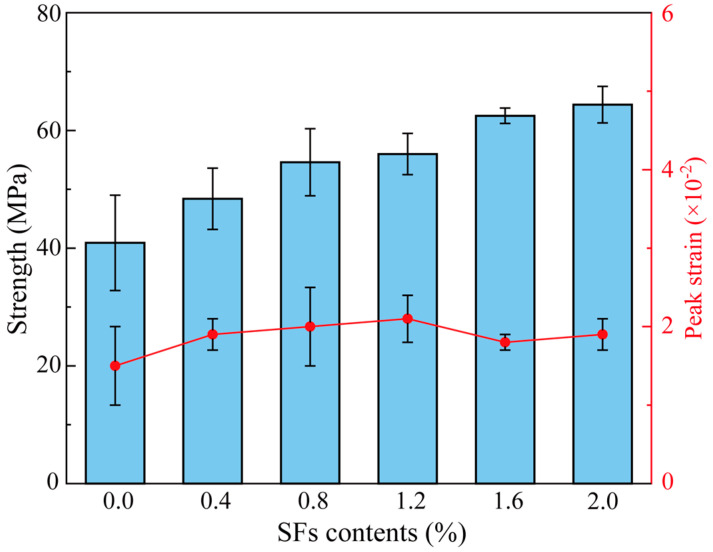
Compressive strength and peak strain of samples with various SF contents.

Figure 9 shows that SFs significantly enhance the strength of the gangue-based functional cementitious material, although the increase in strength does not follow a linear trend as fiber content rises. The compressive strengths of specimens with SF content of 0%, 0.4%, and 0.8% are 41.9 MPa, 49.8 MPa, and 55.6 MPa, respectively, exhibiting a nearly linear increase with a growth rate of 32.62%. However, when the SF content exceeds 0.8%, the strength variation tends to stabilize. At an SF content of 1.6%, the strength reaches 63.5 MPa, representing a 14.24% increase compared to the 0.8% SF content. Even with an increase to 2.0%, the strength gains relative to 0.8% are only 17.53%, which is considerably lower than the growth rate observed below 0.8%. Additionally, Figure 9 illustrates the error distribution for each specimen group, showing that increasing SF content effectively reduces error ranges and minimizes variability in strength among specimens within the same group. The average peak strain of SF-CGCC is also depicted in Figure 9. When the SF content increases from 0 to 1.2%, the peak strain rises by 40% (0.015 to 0.021), confirming the effectiveness of steel fibers in enhancing energy absorption capacity through crack-bridging mechanisms. However, exceeding 1.2% SFs leads to a reduction in peak strain, a phenomenon strongly correlated with localized stress concentrations caused by fiber agglomeration.

### 3.2. Effect of Loading Levels on EIS

**Figure 10:**
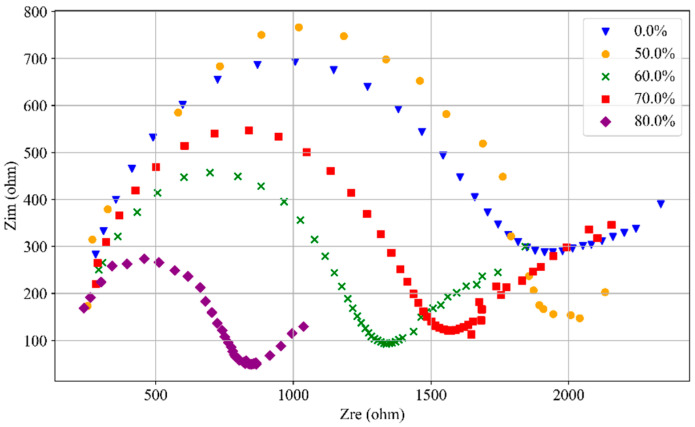
EIS result sample with 0.4% SF content at different loading levels.

The Nyquist curves of SF-CGCC containing 0.4% SFs under different loading levels are shown in Figure 10. The Nyquist plots at other SF contents are also showing a similar kind of variation and, hence, only one graph is represented here. The impedance curves exhibit typical quasi-Randles characteristics [37], featuring a flattened semicircle at high frequencies and a line deviating from 45° in the low-frequency region [38]. The impedance values at the far left of the Nyquist plots are nearly identical, suggesting that damage has minimal impact on the electrolyte solution impedance in the electrochemical system [23]. As the loading force increases, the radius of the semicircular portion in the electrochemical impedance spectra tends to decrease. This indicates that specimen compaction enhances conductive pathways, while steel fiber pull-out reduces resistance within the electrochemical system, ultimately leading to a decrease in impedance [7].

**Figure 11:**
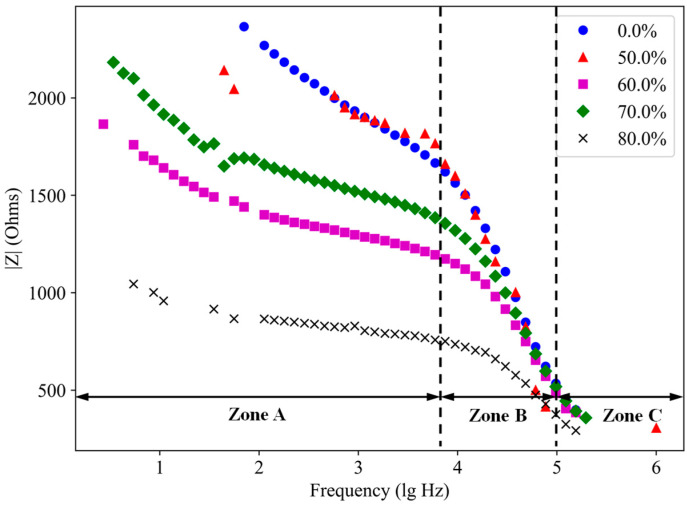
Bode plot of sample with 0.4% SF content at different loading levels.

Figure 11 presents the Bode plots of SF-CGCC containing 0.4% SFs under varying loading levels. The Bode plots at other SF contents are also showing similar kind of variation and hence only one graph is represented here. As shown, the impedance values of SFs-CGCC specimens decrease gradually in Zone A, sharply in Zone B, and then gradually again in Zone C, as frequencies increase from low to high. In the low-frequency range, the impedance values of SF-CGCC specimens are lower than those of the unloaded control specimen. As loading levels increase, specimen impedance decreases significantly. This is because micro-cracks induced by external forces connect the pores within the specimens, creating more conductive pathways and thereby enhancing the electrical conductivity of the SF-CGCC. Conversely, in the high-frequency range (beginning from Zone B), the impedance values of all specimens tend to converge, indicating that the loading forces primarily affect conductivity rather than capacitive properties.

### 3.3. Effect of SF Content on EIS

**Figure 12:**
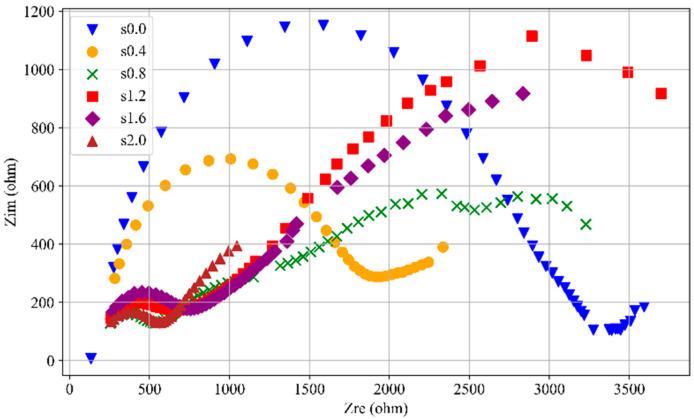
Nyquist plot of samples with different SF contents at 0 loading level.

Figure 12 presents the Nyquist plot for CGCC specimens with varying SF content in an unloaded state. All specimens display the characteristic Randles impedance spectrum. The impedance at the far left of the Nyquist plot remains nearly identical across specimens, indicating that variations in SF content have minimal effect on the conductivity of the internal electrolyte solution. As the SF content increases, the semicircle radius progressively decreases, indicating a continuous reduction in resistance within the specimens’ electrochemical system. This reduction is due to the excellent conductivity of SFs, which create more conductive pathways within the specimens, thereby lowering overall resistance.

Furthermore, when the content of SFs increases to over 0.4%, the Nyquist plot of SF-CGCC exhibits a “dual-arc effect”. This suggests that the fibers introduce two types of conductive paths within the SF-CGCC: one path results from direct contact between SFs, while the other is due to the capacitive effects of SFs positioned in close proximity.

**Figure 13:**
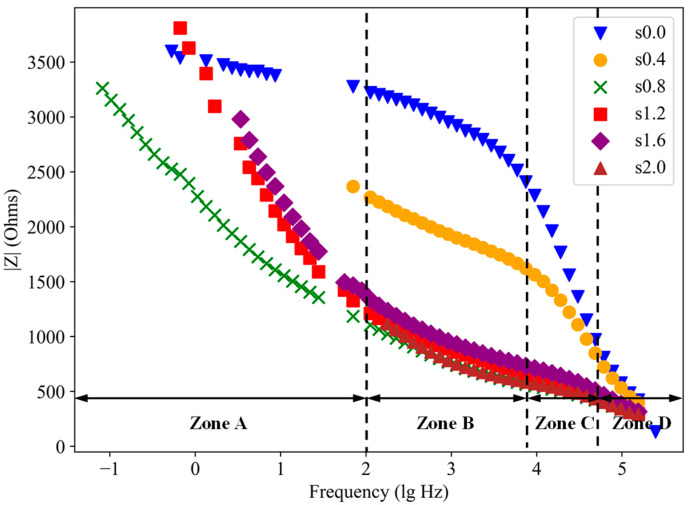
Bode plot of samples with different SF contents at 0 loading level.

Figure 13 shows the Bode plot of samples with varying SF content. Increasing SF content has both positive and negative effects on the conductivity of the specimens. Previous studies have found that conductivity initially increases with added SF content, but then decreases as the SF content continues to rise [39]. This behavior is due to the formation of conductive pathways and the introduction of micropores within the specimens as SFs are added. In the low-frequency range (Zone A), the impedance of specimens with varying SF content exhibits significant variation, as the low-frequency response is primarily governed by the charge transfer resistance [40]. In Zone B, the impedance of specimens exceeding 0.8% SF content tends to converge. This phenomenon occurs because the high-frequency impedance is predominantly determined by matrix resistivity, whereas the influence of the SF content diminishes [41]. Overall, the impedance values of SF-CGCC specimens decrease with increasing SF content. Notably, the specimen containing 0.8% SFs demonstrates lower impedance than that with 1.6% SFs. This trend suggests that excessive SFs incorporation may disrupt conductive pathways within the composite, likely due to interfacial defects or agglomeration-induced electron scattering at higher fiber loadings. In Zone C, the impedance of specimens with varying SF content exhibits a significant difference. This suggests that the influence of SF content on the conductivity of CGCC primarily affects the direct conductive pathways rather than the capacitance.

### 3.4. Analysis of Electrochemical Impedance Parameters

**Figure 14:**
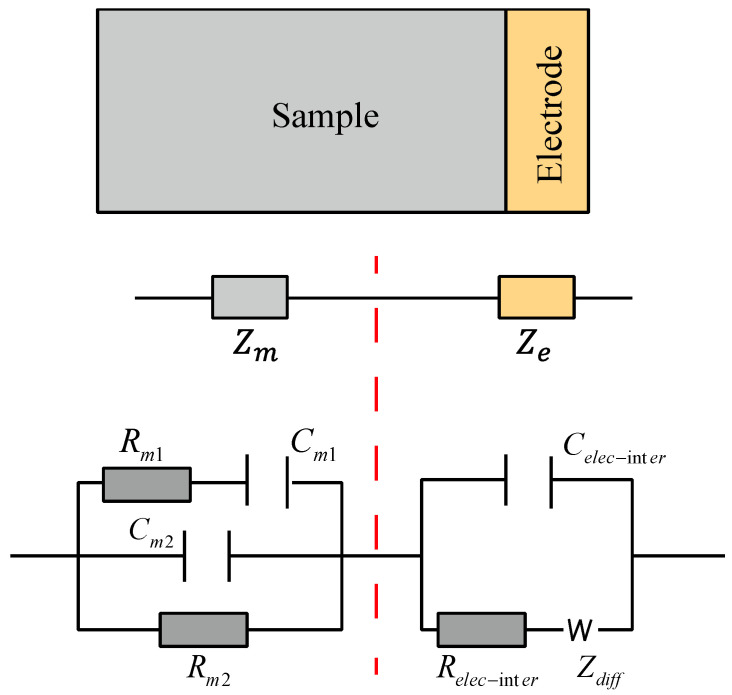
Equivalent circuit model.

The internal structure of the SF-CGCC, as depicted in Figure 14, can be classified as continuous, discontinuous, and insulating paths. An equivalent circuit model proposed by Song et al. [21], also shown in Figure 14, is used to represent this structure and is denoted as ((*R*_m1_*C*_m1_)*C*_m2_*R*_m2_)(*C*_elec-inter_(*R*_elec-inter_*Z*_diff_)) [25,42]. In this model, (*C*_elec-inter_(*R*_elec-inter_*Z*_diff_)) corresponds to the electrochemical reaction occurring between the specimens and the electrodes, which are not related to the microstructures of the SF-CGCC. Meanwhile, ((*R*_m1_*C*_m1_)*C*_m2_*R*_m2_) represents the internal electrochemical reactions within the SF-CGCC, where (*R*_m1_*C*_m1_) denotes the discontinuous paths, *C*_m2_ represents the insulated paths, and *R*_m2_ is the continuous paths. Consequently, the total frequency-dependent impedance *Z*(*ω*) of the entire system can be expressed as follows:

(1) $$Z\left(\omega \right)=Z_{m}+Z_{e}$$

where *Z_m_* and *Z_e_* represent impedance of the CGCC matrix and the electrode interface, respectively, given as follows:

(2) $$Z_{m}=\frac{1}{\frac{1}{R_{m2}}+\frac{1}{R_{m1}+\frac{1}{j\omega C_{m1}}}+j\omega C_{m2}}$$

(3) $$Z_{e}=\frac{R_{\mathrm{ele}\text{-}\mathrm{inter}}+A_{w}\omega^{-1/2}\left(1-j\right)}{1+j\omega \left(R_{\mathrm{ele}\text{-}\mathrm{inter}}+A_{w}\omega^{-1/2}\left(1-j\right)\right)C_{\mathrm{ele}\text{-}\mathrm{inter}}}$$

where j is the imaginary number, ω is the angular frequency of the sinusoidal signal, and Aw is the Warburg constant.

**Figure 15:**
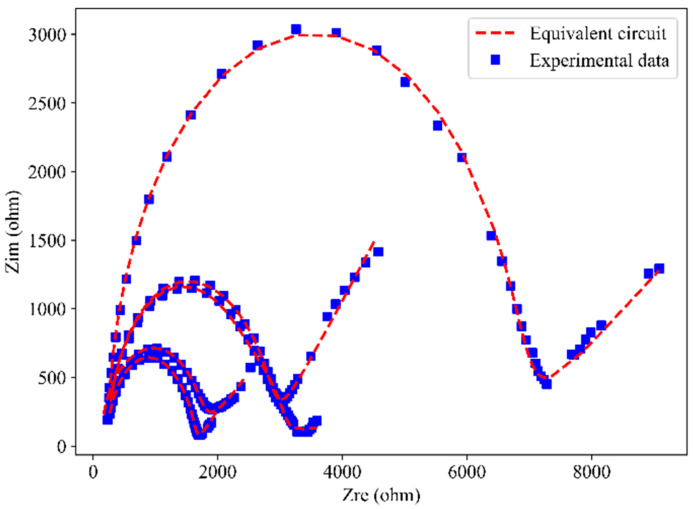
Comparison of equivalent circuit fitted data and experimental data.

Figure 15 presents the Nyquist plots of the experimental data for SF-CGCC content under different conditions, along with the fitted results obtained using the equivalent circuit model. The fitting results align well with the Nyquist curves, accurately representing both the high and low-frequency regions. In Figure 15, the content of SFs is 0.4%. Nevertheless, the aforementioned trends persist for other content.

The values of the impedance parameters *R*_m1_, *C*_m1_, *R*_m2_, and *C*_m2_ of the SFs-CGCC are presented in Table 4.

**Figure 16:**
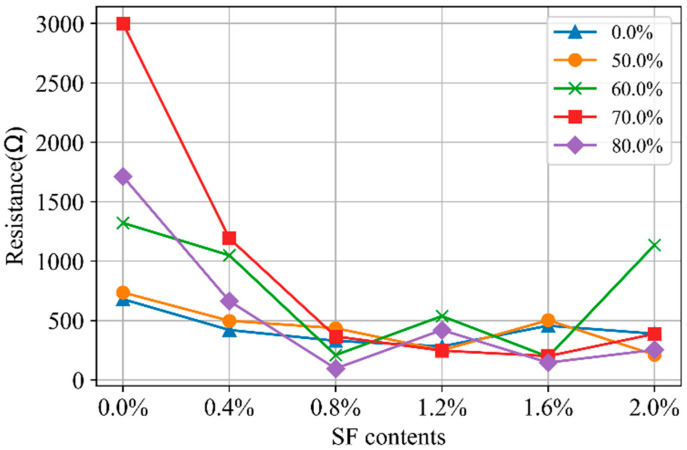
The *R*_m2_ in Nyquist curves for CGCC with different SF contents.

**Figure 17:**
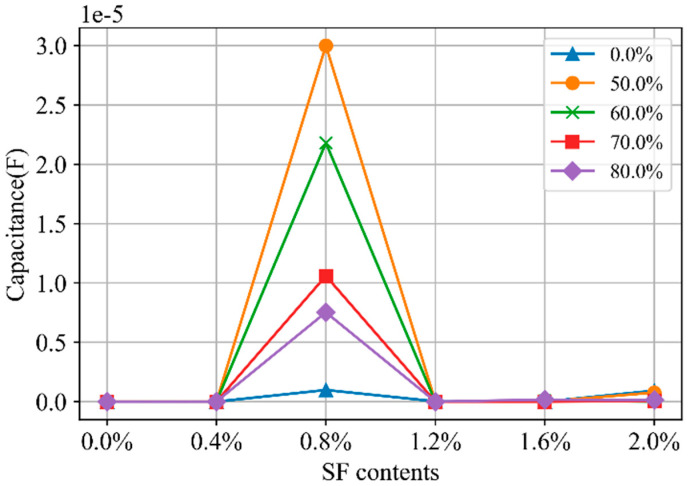
The *C*_m1_ in Nyquist curves for CGCC with different SF contents.

The content of SFs significantly affects the electrochemical impedance of SF-CGCC. Figure 16 illustrates the relationship between *R_m_*_2_ and SF content. As shown, *R_m_*_2_ decreases as the SF content increases from 0 to 0.8%, indicating the formation of more continuous conductive pathways. However, when the SF content rises from 0.8% to 2.0%, the values of *R_m_*_2_ stabilize, suggesting that the addition of SFs beyond 0.8% does not contribute to the creation of new continuous conductive pathways. Figure 17 depicts the relationship between *C_m_*_1_ and the SF content. The capacitance of the discontinuous conductive pathways changes significantly at 0.8% SF content, suggesting that many SFs are in close proximity without direct contact. This leads to an increase in capacitance at the fiber–fiber and fiber–matrix interfaces. However, when the SF content exceeds 0.8%, most of the fibers make direct contact, resulting in a decrease in capacitance.

**Figure 18:**
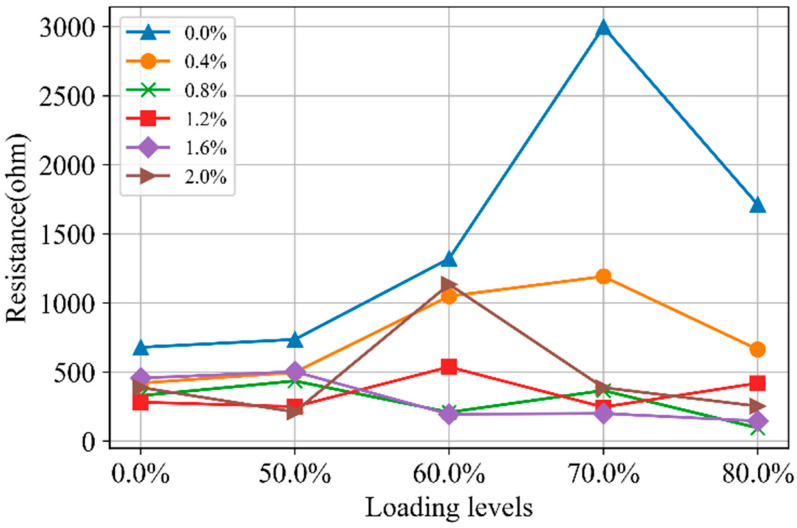
The *R*_m2_ in Nyquist curves for CGCC with different loading levels.

Figure 18 shows the relationship between *R_m_*_2_ and loading levels. The impact of loading levels on the continuous conductive pathways is evident. As the loading level increases from 0 to 70% of strength, the *R_m_*_2_ increases. This is due to the formation of microcracks, which disrupt direct conductive pathways previously established through the pores. However, when the loading level exceeds 70%, the *R_m_*_2_ decreases, as some microcracks connect and form a new continuous conductive pathway.

In the case of SF-CGCC specimens, *R_m_*_2_ remains stable across different loading levels. This suggests that continuous conductive pathways in these specimens are primarily formed by SFs themselves, making them unaffected by microcrack development. However, in the Nyquist plot for specimens with varying SF content (Figure 10), the total resistance decreases, suggesting that compaction primarily influences the conductive pathways formed by pores.

**Figure 19:**
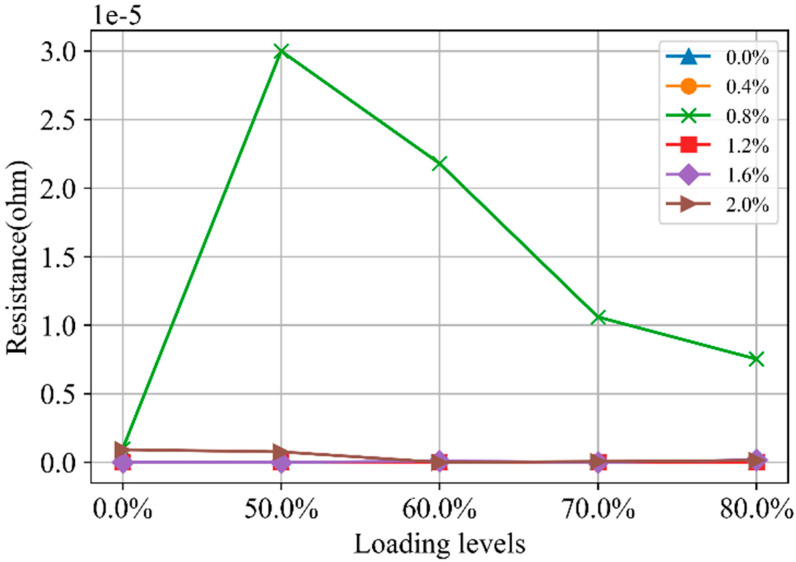
The *C*_m1_ in Nyquist curves for CGCC with different loading levels.

The relationship between *C*_m1_ and loading levels was illustrated in Figure 19. For specimens with SF content other than 0.8%, the capacitance decreases as the loading level increases from 0 to 60% of strength, then stabilizes. This is because the discontinuous conductive pathways are transformed into insulating parts due to the formation of microcracks. In contrast, for specimens with 0.8% SF content, the capacitance primarily arises from the SFs. As the loading level increases from 0 to 50% of strength, compressive deformation reduces the distance between SFs, thereby increasing capacitance. However, when the loading level rises from 50% to 80% of strength, microcrack development begins to hinder the capacitive effect of the SFs.

## 4. Conclusions

This paper proposed a method to evaluate the self-sensing behavior of SF-CGCC using the EIS method. Based on the results, the inclusion of SFs enhances both mechanical properties and conductivity, improving self-sensing capabilities, while the EIS can be used to monitor the mechanical characteristics of SFs-CGCC. The following conclusions were drawn:

1. The mechanical test results demonstrated that the compressive strengths of CGCC specimens showed a nearly linear increase with a growth rate of 32.62% as SF content increased from 0 to 0.8%. Beyond 0.8%, the increase in compressive strength stabilized. In addition, as SF content increases, the post-peak stress–axial strain curve becomes less steep.
2. For SF-CGCC specimens loaded by different external pressures, the conductive pathways induced by SFs remain stable, while the conductive pathways induced by pores increase because of compaction, which results in a decrease of total resistance in the Nyquist plot. Except for specimens with 0.8% SF content, the loading levels primarily affect conductivity rather than capacitive properties.
3. For SFs-CGCC specimens with varying SF content, resistance decreased as SF content increased. Beyond 0.8% SF content, changes in continuous conductive pathways stabilized, indicating that further SF additions did not significantly enhance conductivity.
4. At 0.8% SF content, the fibers formed a preferable conductive network, showing better sensitivity to external forces and satisfactory strength. This suggests that 0.8% SF content optimizes both self-sensing performance, mechanical strength, and material efficiency.

## Figures and Tables

**Table 1:** XRF result sheet for CGAs.

Material	SiO_2_	Al_2_O_3_	Fe_2_O_3_	K_2_O	CaO	TiO_2_	MgO	Na_2_O	SO_5_	Others
CGA/%	62.113	28.169	3.508	2.231	1.224	0.981	0.667	0.400	0.379	0.328

**Table 2:** Main physical properties of CGA.

Density (kg/m^3^)	Water Absorption (%)	Apparent Gravity	Specific Gravity
2850	1.882%	2.631579	2.507375

**Table 3:** Mix proportion of SF-CGCC.

Mix	Mixture Proportion (kg/m^3^)					
	CGA	Cement	Silica Fume	WR	Water	SFs
S0.0	1409.48	563.79	56.38	5.64	253.71	0.00
S0.4	1403.84	561.54	56.15	5.62	252.69	31.40
S0.8	1398.21	559.28	55.93	5.59	251.68	62.80
S1.2	1392.57	557.03	55.70	5.57	250.66	94.20
S1.6	1386.93	554.77	55.48	5.55	249.65	125.60
S2.0	1381.29	552.52	55.25	5.53	248.63	157.00

Note: WR refers to Water reducer.

**Table 4:** Fitting results of EIS data.

Mix	Loading Level	*R_m_*_1_ (Ω)	*C_m_*_1_ (nF)	*R_m_*_2_ (Ω)	*C_m_*_2_ (pF)
S0.0	0.0	178.9	5.40	679.3	7.00
	0.5	158.2	3.77	736	15.60
	0.6	384.9	2.48	1321	446.00
	0.7	341.7	3.83	2998	549.00
	0.8	384.6	2.93	1712	519.00
S0.4	0.0	306.6	2.63	420.2	18.80
	0.5	223.8	3.44	498	283.00
	0.6	375.1	2.50	1049	344.00
	0.7	416.4	2.43	1192	484.00
	0.8	317.9	2.99	664	372.00
S0.8	0.0	328.8	982.00	329.2	0.00
	0.5	357.7	3.00 × 10^4^	435.7	0.00
	0.6	718.5	2.18 × 10^4^	209	0.00
	0.7	498.5	1.06 × 10^4^	366.6	233.00
	0.8	2.13 × 10^7^	7.53 × 10^3^	95.59	5.54 × 10^7^
S1.2	0.0	984.1	29.30	282.6	576.00
	0.5	986.3	18.10	249.4	0.00
	0.6	363.4	3.02	537.2	96.20
	0.7	361.8	2.28	246.7	0.00
	0.8	299.8	3.27	420	278.00
	0.9	332.6	3.28	423.8	0.00
S1.6	0.0	510.8	2.55	456.4	560.00
	0.5	462.8	2.50	502.7	424.00
	0.6	1981	94.60	194.8	346.00
	0.7	6826	0.00	200.5	386.00
	0.8	694.2	173.00	145.8	0.00
	0.9	529.6	2.47	433	560.00
S2.0	0.0	383.3	925.00	388.8	0.01
	0.5	558.4	765.00	211	18.70
	0.6	423	2.64	1135	600.00
	0.7	1188	62.90	387.4	1810.00
	0.8	508.7	147.00	253.2	608.00
	0.9	1022	5.84	164.7	0.00

## Data Availability

The original contributions presented in this study are included in the article. Further inquiries can be directed to the corresponding author.
